# Nutritional adequacy of a novel human milk fortifier from donkey milk in feeding preterm infants: study protocol of a randomized controlled clinical trial

**DOI:** 10.1186/s12937-017-0308-8

**Published:** 2018-01-09

**Authors:** Alessandra Coscia, Enrico Bertino, Paola Tonetto, Chiara Peila, Francesco Cresi, Sertac Arslanoglu, Guido E Moro, Elena Spada, Silvano Milani, Marzia Giribaldi, Sara Antoniazzi, Amedeo Conti, Laura Cavallarin

**Affiliations:** 1 Neonatal Unit of Turin University, City of Health and Science of Turin, Via Ventimiglia 3, 10126 Turin, Italy; 2 Italian Association of Human Milk Banks, Via Libero Temolo 4, 20126 Milan, Italy; 3 Unit of Medical Statistics and Biometry, Department of Clinical Sciences and Community Health, University of Milan, Milan, Italy; 4 Institute of Sciences of Food Production, National Research Council, Largo Braccini 2, 10095 Grugliasco (TO), Italy; 5 Research Centre for Engineering and Agro-Food Processing, Council for Agricultural Research and Economics (CREA), Strada delle cacce 73, 10135 Turin, Italy

**Keywords:** Human milk, Human milk fortifier, Donkey milk, Adjustable fortification, VLBW infants, Preterm infants, Enteral feeding, Feeding intolerance

## Abstract

**Background:**

Fortification of human milk is a standard practice for feeding very low birth weight infants. However, preterm infants often still experience suboptimal growth and feeding intolerance. New fortification strategies and different commercially available fortifiers have been developed. Commercially available fortifiers are constituted by a blend of ingredients from different sources, including plant oils and bovine milk proteins, thus presenting remarkable differences in the quality of macronutrients with respect to human milk. Based on the consideration that donkey milk has been suggested as a valid alternative for children allergic to cow’s milk proteins, due to its biochemical similarity to human milk, we hypothesized that donkey milk could be a suitable ingredient for developing an innovative human milk fortifier.

The aim of the study is to evaluate feeding tolerance, growth and clinical short and long-term outcomes in a population of preterm infants fed with a novel multi-component fortifier and a protein concentrate derived from donkey milk, in comparison to an analogous population fed with traditional fortifier and protein supplement containing bovine milk proteins.

**Methods:**

The study has been designed as a randomized, controlled, single-blind clinical trial. Infants born <1500 g and <32 weeks of gestational age were randomized to receive for 21 days either a combination of control bovine milk-based multicomponent fortifier and protein supplement, or a combination of a novel multicomponent fortifier and protein supplement derived from donkey milk. The fortification protocol followed is the same for the two groups, and the two diets were designed to be isoproteic and isocaloric. Weight, length and head circumference are measured; feeding tolerance is assessed by a standardized protocol. The occurrence of sepsis, necrotizing enterocolitis and adverse effects are monitored.

**Discussion:**

This is the first clinical study investigating the use of a human milk fortifier derived from donkey milk for the nutrition of preterm infants. If donkey milk derived products will be shown to improve the feeding tolerance or either of the clinical, metabolic, neurological or auxological outcomes of preterm infants, it would be an absolute innovation in the field of feeding practices for preterm infants.

**Trial registration:**

ISRCTN -ISRCTN70022881.

## Background

Very preterm newborns (gestational age < 32 weeks) and Very Low Birthweight Infants (VLBWI, birthweight <1500 g) currently represent the majority of patients assisted in Neonatal Intensive Care Units (NICU) [1]. The increase of the survival rate for these newborns, due to improvements in perinatal care, has opened new perspectives regarding their outcome and has a significant impact on their health status in adulthood.

Although very preterm or VLBWI usually attain some “catch-up” growth following hospital discharge, growth deficits can persist throughout childhood and adolescence, and into adulthood. Slow postnatal growth is associated with neurodevelopmental impairment in later childhood, with poorer cognitive outcomes, and may have consequences for long-term metabolic and cardiovascular health [2–4]. Nutrition represents a fundamental factor for long-term survival and quality of life for this group of infants. The main issue is to ensure an adequate qualitative and quantitative nutrition, particularly in terms of protein intake, which is the main cause of post-natal growth deficit.

Human milk is the recommended form of enteral nutrition for all neonates including preterm infants [5]. Breast milk alone, however, does not meet the recommended nutritional needs for growth in preterm infants [6, 7]. The most common strategy in neonatal care facilities is to cope with these potential nutrient deficits by supplementing breast milk with additional nutrients (mainly proteins and minerals) to ensure a sufficient caloric intake in consideration of the special nutritional requirements [8–10]. The fortification of human milk still represents a significant challenge [11, 12]. The standard fortification strategies have been considered unsatisfactory to sustain an appropriate growth. Thus, new approaches were developed, among which Adjustable (ADJ) Fortification currently seems to be the most promising [13]. It involves the use of a protein supplement on an individualized basis, in addition to a multi-component fortifier. This method, accepted at an international level, consists of providing a variable protein intake based on the metabolic response of each single newborn by titration to the infant’s blood urea nitrogen level [14, 15]. This approach requires simple, high-quality and well-tolerated protein supplements which, unfortunately, are not readily available. Most commercially available multi-nutrient fortifiers are derived from bovine milk, which has a protein composition very different from that of human milk. Moreover, cow milk protein intake in the first months of life has raised concerns because of its association with allergies [16] and some studies have observed a possible role of cow’s milk proteins as a trigger of intestinal inflammation in preterm neonates. [17] Investigations on exclusive HM diets (human milk-based fortifier and donor HM, if mother’s milk unavailable) have been carried out in recent years. All these studies recently underwent to a systematic review [18]; most of them were retrospective studies and not randomized clinical trials. Authors conclude that there is not strong evidence that human milk based fortifiers in otherwise exclusively human milk-fed preterm infants affect important outcomes. Consistently, milk from mono-gastric animals, rather than from ruminants, has been suggested during recent years to be more suitable for human nutrition based on their physiochemical properties [19]. In previous studies, our group observed on children affected by cow milk protein allergy that donkey milk (DM) was highly tolerated [20], and found that its protein and lipid fractions showed a substantial similarity to that of human milk [21, 22]. DM has an n-3 PUFA (polyunsaturated fatty acids) content equivalent to human milk, and is rich in lysozyme, a protein characterized by antibacterial properties, able to hinder pathogen growth, and milk spoilage. It has been recently demonstrated in murine models that a supplementation of the basal diet with DM decreases the accumulation of body lipids and affects glucose and lipid metabolism in a manner more similar to human milk than cow milk [23] These biological effects resulted comparable with those elicited by human milk [24] Based on the above considerations, it can be speculated that DM is more suitable than bovine milk to be an ingredient of a human milk fortifier for VLBW Infants and preterm newborns.

Our hypothesis is that feeding these newborns according to ADJ fortification principles, with human milk fortified by protein and multi-component supplements derived from DM, will improve the feeding tolerance and the clinical, metabolic, neurological and auxological outcome at short- and long-term.

We present the protocol of a study aimed to evaluate the use of DM-derived multi-component fortifier and protein concentrate for the nutrition of infants with birthweight <1500 g or gestational age < 32 weeks. This evaluation is being performed through a clinical trial (randomized, controlled, blind) by comparing it with commercial bovine milk-based multi-component fortifier and protein concentrate.

## Methods/design

The study is currently underway at the Neonatal Intensive Care Unit (NICU) of the University of Turin, and has been approved by Local Ethic Committee. Informed written consent is obtained from parents before enrollment. The trial was registered on ISRCTN Registry BioMed Central (Registration number: ISRCTN70022881).

### Study population

All patients admitted to our unit that met the following inclusion criteria were consecutively enrolled in the study.

Inclusion criteria:

- Gestational age < 32 weeks or birthweight <1500 g
- Exclusive feeding with human milk (fresh own mother’s or donor milk)
- Human milk volume > 80 ml/kg/day within the first 4 weeks of life

Exclusion criteria:

- Severe gastrointestinal pathologies (diagnosed or suspected necrotizing enterocolitis, colostomy, intestinal obstruction, symptoms of peritonitis, presence of blood in the feces)
- Chromosomal abnormalities or major malformations
- Hereditary metabolic diseases
- Intravascular disseminated coagulopathy (IDC), shock
- Patent Ductus Arteriosus (PDA) requiring medical care or surgery at time of randomization
- Severe renal failure (serum creatinine >2 mg/dl)

### Study design

Infants meeting inclusion criteria are identified and their parents approached for consent. After informed written parental consent is obtained, infants are randomized 1:1 by a software-generated list in one of the following groups:

- Control group: Adjustable Fortification with commercial multi-component fortifier (FM85 Nestlè) and protein concentrate (Protifar Nutricia), named BMC and BPC respectively, derived from bovine milk, for a minimum of 21 days (if necessary, fortification is continued after this period using the same type of product)
- Fortilat group: Adjustable Fortification with multi-component fortifier and protein concentrate derived from donkey milk, named DMC and DPC respectively, not commercially available, and prepared according to current EU legislation on Foods for special medical purposes, for a minimum of 21 days (if necessary, fortification is continued after this period using the same type of product).

The composition of BMC and DMC are provided in Table 1. Table 2 presents the composition of BPC and DPC. The experimental products were produced by ultrafiltration of pasteurized donkey milk in a pilot stainless steel plant. Retentates from the ultrafiltration processes were then pasteurized and aseptically lyophilized and packed. All the batches used for the trial were analyzed for the microbiological and chemical profile and complied with the safety criteria required by Italian legislation. The products are stored at −80 °C until used.

**Table 1:** Macro-composition of the multi-component fortifiers derived from bovine milk (BMC) and from donkey milk (DMC). Values per 100 g of product

	BMC	DMC
Protein g (Nx6.25)	20.0	22.5
Carbohydrate g	66.0	59.0
of which:		
Lactose g	6.0	59.0
Maltodextrine g	60.0	0.0
Fat g	0.4	3.6
Energy:		
Kcal	385	390
kcal/g protein	18.8	15.6
Calcium mg	1500	938
Phosphate mg	900	734
Osmolality mOsm/Kg	453	441

**Table 2:** Composition of the protein concentrates derived from bovine milk (BPC) and from donkey milk (DPC). Values per 100 g of product

	BPC	DPC
Protein g (Nx6.25)	88.5	43
Carbohydrate g	<1.5	33.5
Fat g	≤2.0	6.1
Energy Kcal	370	418
Calcium mg	1350	1650
Phosphate mg	700	1150

A nutrition protocol following the criteria of ADJ fortification is being followed to ensure that fortification advancement is consistent for all study participants. Tables 3 and 4 show the ADJ Fortification criteria for the control products (BMC and BPC) and for the test products (DMC and DPC). Since the protein concentration and energy content of the bovine milk based products differ from the donkey milk based products, the amounts of powder required to obtain the same level of fortification are different depending on the product in use (Table 4). Because the same nurses in charge of feeds preparation and administration are also in charge of evaluating signs of feeding tolerance, it is not possible to achieve a double blindness of the intervention. As caregivers are aware of the group allocation of infants, this is to be defined as a single-blind randomized controlled trial.

**Table 3:** Adjustable Fortification criteria based on weekly blood urea nitrogen (BUN) values

BUN (blood urea nitrogen)	
< 10 mg/dl	Increase fortification by one level
10–16 mg/dl	No change
> 16 mg/dl	Decrease fortification by one level

**Table 4:** Amount of fortifer and protein supplement at the various fortification levels, according to ADJ fortification protocol

	Multicomponent			Protein concentrate	
Levels	BMC g/ml	DMC g/ml		BPC g/ml	DPC g/ml
2.5%	0.025	0.02		–	–
4%	0.04	0.032		–	–
5%	0.05	0.04		–	–
5% +1	0.05	0.04	+	0.004	0.008
5% +2	0.05	0.04	+	0.008	0.016
5% +3	0.05	0.04	+	0.012	0.024

Advancing of enteral feeds is strictly regulated according to the feeding protocol adopted in our NICU, based on the evaluation of signs of feeding intolerance (available on demand to the authors). The criteria for hospital discharge are uniform, i.e., satisfactory weight gain while receiving full oral feeding, maintenance of adequate thermal stability and resolution of acute medical conditions.

### Outcome measures

1. Primary Endpoint.
  Occurrence of at least one episode of feeding intolerance, defined as interruption of enteral feeding for at least eight consecutive hours during the observation period.
2. Secondary Endpoints.
  2.1 Gastrointestinal outcomes: Number of feeding intolerance episodes, number of feeding interruption episodes (of any duration), total hours of enteral feeding interruption, time required to reach full enteral feeding (150 ml/kg/day).
  2.2 Gastric Emptying time (ultrasonic measurements of the antral cross sectional area) [25, 26]: half-time of gastric emptying (minutes) and time of gastric emptying (minutes).
  2.3 Esophageal impedance and pH monitoring (MII/pH) [27, 28]: GER frequency (reflux events/h), bolus reflux extent (number of channels), Bolus clearance time (seconds), bolus exposure index and reflux index (percentage).
  2.4 Clinical outcomes: necrotizing enterocolitis, suspected or confirmed sepsis, mortality, hospital stay duration, intraventricular hemorrhage, retinopathy of prematurity (defined according to the Vermont Oxford Network) [29].
  2.5 Metabolic and auxological outcomes (as shown in the synoptic table) (Table 5).

**Table 5:** Synoptic table. T0: before starting the fortification; T1: day 7 after beginning the fortification (if level 3 of ADJ fortification) or as soon as level 3 is reached; T2: day 14 since beginning of fortification; T3: day 21 since beginning of fortification

	STUDY PERIOD						
	Enrolment	Allocation	Post-allocation				Close-out
Timepoint			T0	T1	T2	T3	
Enrolment:							
Eligibility screen	X						
Informed consent	X						
Full patient history collection	X						
Allocation		X					
Intervention:							
BUN			X	X	X	X	
Albumin			X	X	X	X	
Creatinine			X	X	X	X	
Calcium			X	X	X	X	
Phosphate			X	X	X	X	
Alkaline Phosphatase			X	X	X	X	
Weight			X	X	X	X	
Lenght			X	X	X	X	
Head circumference			X	X	X	X	
Plasma Aminoacids			X			X	
Urine Aminoacids			X			X	
Urinary Metabolomic Profile			X			X	
Calprotectin			X			X	
pH			X	X	X	X	
BE			X	X	X	X	
HCO_3_^−^			X	X	X	X	
MII/pH						X	
Gastric emptying time						X	
Assesments							
List other data variables							X
List other data variables							X

### Sample size

The sample size has been determined based on the occurrence of primary endpoint (at least one episode of interruption of enteral feeding ≥8 h). Based on the data available in our NICU from the population of VLBWI or preterm infants, about 45% of infants present at least one interruption of enteral feeding ≥8 h.

In the hypothesis that the use of a better tolerated fortifier causes a 25% reduction in the frequency of the primary endpoint, and setting the risk of type I errors and the power at the usual 5 and 80%, 62 newborns per group have been considered as required.

### Planned recruitment rate and compliance

In the year 2014, 115 infants less than 32 weeks gestation and weighting less than 1500 g were admitted to our NICU. Breastfeeding initiation rates with mothers’ own milk or donor milk were around 90% in our nursery. Therefore, around 100 infants per year were estimated to be eligible for the study, with a recruitment rate higher than 60%. We did not anticipate problems with compliance. The majority of mothers visits their baby on a regular basis, hence they are easily approached for consent as soon as the infant reaches the point to start fortification. Blood sampling required by the study protocol is consistent with the routine monitoring performed in our unit, hence there is no supplementary inconvenience for the patients and it is easily accepted by parents.

### Data collection methods

In order to promote the data quality, it has been provided a training plan for the nurses and clinicians based on fortification protocols and evaluation of feeding tolerance. Data are being collected according to the scheme shown in the synoptic table of the study (Table 5). Weight, length and head circumference measurements are recorded at birth, and during the observation period (weight: daily; length and head circumference: weekly), at 36 ± 1 weeks of postmenstrual age, 40 ± 1 weeks of postmenstrual age, at 6, 12 and 18 months of corrected age. The collection of outcomes, baseline, and other trial data are reported in the specific form by copying them from the medical record at discharge of the newborn.

### Estimated rate of loss to follow up

Our NICU is a tertiary level neonatal unit. Most babies in the NICU stay until discharge from hospital. Very few infants return to local hospitals prior to discharge home, and it generally is very rare that they are transferred prior to 35 weeks corrected gestational age. In addition, all infants that meet inclusion criteria for the study are planned to be followed until two years of age, as part of our follow-up program. We therefore do not expect any significant loss to follow up.

### Statistical analysis

The statistical analysis will be performed by the Medical Statistics and Biometry Unit of the Clinical and Community Sciences of the University of Milan.

At the end of the recruitment, the occurrence of the primary endpoint will be evaluated on the intention-to-treat population (all randomized infants). The comparison between the two study groups will be performed with the exact Fisher test. All secondary endpoints will be modelled in the framework of generalised linear model [30] allowing, when appropriate, for relevant covariates. The number of episodes of feeding intolerance (0, 1 … k) will be modelled as a Poisson variable. Total hours of enteral feeding interruption, time required to reach full enteral feeding, hospital stay duration, gastric emptying time and MII/pH parameters will be modelled (after proper scale transformation, if required) as normal variables. The occurrence of clinical outcomes, assesed as dychotomous variable (yes/no) will be modelled as binomial variables. Metabolic and auxological outcomes, which are repeatedly during study period, will be modelled (after proper scale transformation, if required) as normal variables, with a model allowing for repeated measures. All statistical models are fit with SAS software [31].

## Discussion

We present the protocol of a study aimed to assess the effects of a new human milk fortifier derived from donkey milk on VLBWI feeding tolerance and growth. To the best of our knowledge, this trial is unique, being the first study that investigates the use of a human milk fortifier derived from donkey milk for the nutrition of preterm and VLBWI.

In our trial all the infants received exclusively human milk (Fresh own mother’s milk or Pasteurized donor milk) without any preterm formula supplementation. The trial of Sullivan, comparing a human milk based fortifier with a bovine milk based fortifier, included in the group supplemented with the bovine fortifier also subjects fed with preterm formula [32]. This represents a confounding variable masking the effects of the two different fortifiers as a sole supplement of human milk.

If the donkey milk derived fortifier will be proved to improve the feeding tolerance or some of the other outcomes (clinical, metabolic, neurological or auxological) of very preterm and VLBWI, it would be an absolute innovation in the field of feeding practices for preterm infants.

A possible limitation of our study is that it has been designed as a single-blind randomized controlled trial, being the caregivers aware of infants’ allocation in the study. To try to eliminate any possible bias, a protocol about the evaluation of the signs of feeding intolerance has been introduced in our unit before the beginning of the study. A strict adhesion to the protocol was recommended to all the nursing staff.

## Abbreviations

ADJ: Adjustable Fortification
BMC: Multi-component fortifiers derived from bovine milk
BPC: Protein concentrates derived from bovine milk
BUN: Blood urea nitrogen
DMC: Multi-component fortifiers derived from donkey milk
DPC: Protein concentrates derived from donkey milk
GER: Gastroesophageal Reflux
MII/pH: Multichannel Intraluminal Impedance and pH monitoring
NICU: Neonatal Intensive Care Unit
SDS: Standard deviation score
VLBWI: Very Low Birthweight Infants
